# Cost of Care for Newborns With Neonatal Abstinence Syndrome in a State Medicaid Program

**DOI:** 10.1001/jamanetworkopen.2024.0295

**Published:** 2024-02-26

**Authors:** Caroline Jenkins, Matthew Hudnall, Courtney Hanson, Dwight Lewis, Jason M. Parton

**Affiliations:** 1 Institute of Business Analytics, The University of Alabama, Tuscaloosa; 2 Institute of Data and Analytics, The University of Alabama, Tuscaloosa

## Abstract

**Importance:**

Neonatal abstinence syndrome (NAS) is a medical condition among neonates experiencing substance withdrawal due to the mother’s substance use during pregnancy. While previous studies suggest that the overall incidence and annual costs of NAS are increasing, to date, the long-term costs have yet to be demonstrated in Medicaid populations.

**Objective:**

To examine the demographic differences and long-term costs of care for neonates diagnosed with vs not diagnosed with NAS.

**Design, Setting, and Participants:**

This cohort study used claims data from the Alabama Medicaid Agency for neonates born to Medicaid-eligible mothers between January 1, 2010, and December 31, 2020. Data were analyzed in June 2022.

**Exposure:**

A diagnosis of NAS within 30 days of birth.

**Main Outcomes and Measures:**

Rate of NAS by demographic and birth characteristics, long-term costs attributable to NAS status and demographic and birth characteristics, and distribution of this expenditure over the enrollment period.

**Results:**

A total of 346 259 neonates with Medicaid eligibility were born during the study period (mean [SD] gestational age, 38.4 [2.2] weeks; 50.5%, male), 4027 (1.2%) of whom had an NAS diagnosis within 30 days of birth. A larger percentage of neonates with an NAS diagnosis were male (52.7%) than in the group without NAS (50.5%). Neonates with NAS also weighed less at birth (mean difference, −212.0 g; 95% CI, −231.1 to −192.8 g) and had older mothers (mean difference, 3.4 years; 95% CI, 2.6-4.2 years). An NAS diagnosis had an estimated additional cost of $17 921 (95% CI, $14 830-$21 012) over the enrollment period, and this cost was not evenly distributed over that period.

**Conclusions and Relevance:**

In this cohort study of neonates born into the Alabama Medicaid population, those with an NAS diagnosis had a different demographic profile and a higher cost to state Medicaid agencies than those without NAS. These findings warrant further effort to reduce the occurrence of NAS.

## Key Points

**Question:**

What is the cost of care for neonates born to Medicaid-eligible mothers who are diagnosed with neonatal abstinence syndrome (NAS) within 30 days of birth?

**Findings:**

In this cohort study of 346 259 neonates born eligible for Alabama Medicaid, those with an NAS diagnosis within 30 days of birth had estimated long-term health care costs $17 921 higher than those without an NAS diagnosis.

**Meaning:**

The findings suggest that the cost of care for individuals born with NAS places a significant burden on state Medicaid agencies and calls for the mitigation of opioid use in expectant mothers.

## Teaser

This cohort study estimates 10-year costs of care for neonatal abstinence syndrome in a neonates with Medicaid-eligible mothers in Alabama.

## Introduction

Neonatal abstinence syndrome (NAS) is a withdrawal diagnosis experienced by neonates following substance use by the mother during pregnancy. The term *neonatal opioid withdrawal* has also been used but falls under the broader category of NAS and refers to withdrawal symptoms directly correlated with opioid use. Herein, NAS refers to a collection of drug-related withdrawal symptoms involving the central nervous and gastrointestinal systems in newborns. A prior study showed that 98% of neonates with NAS were exposed to opioids in utero.^1^ Neonates with NAS are more likely to have birth complications, including low birth weight, breathing issues, jaundice, feeding difficulties, seizures, and possible sepsis.^2,3^ While not often fatal, NAS generally involves extended hospital stays after birth. Onset is typically within 60 hours after delivery and can last from 2 days to over a month.^4^

The incidence of NAS has been increasing throughout the US.^5^ Incidence in the East South Central US census region (Kentucky, Tennessee, Mississippi, and Alabama) is the highest in the US, with 16.2 births of neonates with NAS per 1000 population as of 2015.^3^ In Medicaid populations, the incidence and cost of births of neonates with NAS are also increasing.^4^ Using 2014 data, Winkelman et al^6^ estimated that NAS-related birth expenditures cost Medicaid about $462 million annually. Corr and Hollenbeak^7^ estimated that these expenditures were $316 million in 2012 and attributed at least some of the increase to increasing incidence rates of NAS. Alabama is 1 of 16 states not currently participating in the Healthcare Cost and Utilization Project, which many recent studies have used to examine NAS costs and incidence rates.^8^

Federal and US state governments handle most of the economic burden associated with NAS, as Medicaid programs financially cover most neonates born with NAS in the US.^3,7^ In Alabama, Medicaid covers over half of deliveries.^9^ The population of neonates in Alabama whose births were paid for by Medicaid has experienced trends analogous to those in the literature, including an increasing number of neonates born with NAS.^4,5^ Additionally, Alabama had the highest rate of opioid prescriptions per capita between 2019 and 2021.^10^

The economic burden of NAS does not immediately dissipate after birth, as associated issues likely have long-term consequences.^10,11^ Neonates diagnosed with NAS in commercial insurance populations are more likely to use health care resources at a higher rate later in life compared with those without NAS.^9^ This increased use may be associated with a long-term cost burden. Furthermore, children with prenatal opioid exposure have more regulatory problems and delays in cognitive functioning than those without.^11,12^

Cost-related NAS inquiries among Medicaid populations have primarily relied on the publicly available Kids’ Inpatient Database (KID), which is not structured for studies with longitudinal designs.^3,7^ Moreover, KID data are only available every 3 years and, therefore, are unsuitable for examining incidence rates at policy-relevant time intervals. A long-term study by Corr et al^13^ analyzed 11 years of data using the Medicaid Analytic eXtract files but had issues similar to those of studies using the KID data,^3,7^ as the most recent year of these data are 2013. On the other end of the spectrum, Liu et al^14^ were able to examine long-term health care use and cost but used data provided by commercial insurance providers to calculate cost ratios.^10^ Their insights offer understanding of the total economic burden of NAS. Still, given that most of this burden lies with state Medicaid populations, many unknowns remain regarding NAS-associated health care costs in the US. Moreover, Medicaid and commercial insurance populations vary significantly in sociodemographic characteristics and opioid prescribing practices for insured recipients.^14,15^

We aimed to estimate the cost of care for neonates born to Medicaid-eligible mothers who were diagnosed with NAS within 30 days after birth. To this end, we considered the costs for individuals born with NAS in Alabama’s Medicaid population from birth to the end of continuous eligibility or the 10th year of life, whichever came first. Additionally, we aimed to estimate the distribution of these financial consequences for Medicaid.

Given the insights of existing literature,^9,10,11^ we hypothesized that health care costs for individuals born with NAS would continue to increase as they aged. While cost differences between individuals born with vs without NAS are smaller in the period following birth than long term, we expected them to differ statistically. Liu et al^14^ discovered that individuals with an NAS diagnosis shortly after birth used health care services at a higher rate through 8 years of age, with costs almost double those for children without an NAS diagnosis. Understanding this actual cost is vital to Medicaid agencies in setting budgets and implementing policy decisions.

## Methods

### Design

We designed this cohort study to investigate the long-term economic burden of a diagnosis of NAS after birth. Methods were chosen to estimate the relative costs of each included variable and to calculate NAS-related costs for individuals born in the study period. Our design was a derivative of the methods used by Corr and Hollenbeak^7^ to estimate health care utilization among children with NAS. Given our similar goal of estimating cost and our equivalent risk of confounding, we also chose to run regression on a matched sample.^15^ Our data were matched using full Mahalanobis propensity score methods on sex, rurality, birth year, Medicaid eligibility months, race and ethnicity, birth weight, gestational age, and mother’s age at delivery. Additionally, we were able to break down these costs by year of life to assess the long-term impact of an NAS diagnosis. The University of Alabama’s institutional review board approved the study and waived the need for informed consent because consent to use data for study purposes is granted as a result of Alabama Medicaid enrollment and data identifiers were removed prior to analysis. The Strengthening the Reporting of Observational Studies in Epidemiology (STROBE) reporting guideline was followed.

### Data Sources

Data for this study came from the Alabama Medicaid Agency’s administrative database. The database includes reimbursed health care claims and sociodemographic information at the individual level between January 1, 2010, and December 31, 2020.

In selecting members to be included in the study, we chose to only include individuals with at least 1 year of eligibility following birth and whose mothers were also enrolled in Medicaid at the time of birth. Linking individuals to their respective mothers provided additional birth and prenatal care information. Additionally, limiting this study to individuals with an associated mother provided a population over which Medicaid programs hold influence.

We obtained NAS status using *International Classification of Diseases, Ninth Revision (ICD-9)* and *International Statistical Classification of Diseases and Related Health Problems, Tenth Revision (ICD-10)* diagnosis codes. These codes included *ICD-9* 7795 (drug withdrawal syndrome in newborns), *ICD-10* P961 (neonatal withdrawal symptoms from maternal use of drugs of addiction), and *ICD-10* P962 (withdrawal symptoms from therapeutic use of drugs in newborns). Individuals were determined to have had an NAS diagnosis if a claim with 1 of these diagnosis codes occurred within 30 days of birth. This cutoff was used because NAS generally persists for less than 1 month.^4^

### Outcomes

The dependent variables of interest in this study were the total incurred cost throughout Medicaid enrollment over the first 10 years of life and the yearly distribution of this expenditure. This included costs related to the initial diagnosis and treatment of NAS. Costs were derived from amounts paid by Alabama Medicaid for claims initiated within 10 years of birth. Payments were adjusted for inflation to 2021 US dollars using the US Bureau of Labor Statistics Consumer Price Index.^16^

### Independent Variables

Sociodemographic and health-related variables were considered in this study. Following the design of Corr et al,^13^ we included sociodemographic and potential confounding factors in our analysis. Sociodemographic factors included sex, race and ethnicity, birth year, and whether the individual lived in a rural or urban environment at birth. Sex was treated as a binary variable. Race and ethnicity, included for consistency with the study by Corr and Hollenbeak,^7^ were self-reported and categorized as Black, Hispanic, White, and other (which included all non-Hispanic races not listed). The birth year and the mother’s age at delivery were treated as continuous variables in the main model. Gestational age at birth, birth weight, and the presence of a previous opioid prescription for the mother were considered factors associated with health. Gestational age (weeks) and birth weight (grams) are indicators of premature birth and could indicate additional health problems later in life. Both were treated as continuous variables. As birth weight data were not available for neonates born in 2014, these neonates were removed in the matching process. The presence of a previous opioid prescription was treated as a binary variable. The number of Medicaid eligibility months was treated as a continuous variable to account for enrollment variation. Birth weight was the only variable with missing data.

### Statistical Analysis

Our statistical analysis plan closely followed that laid out by Corr and Hollenbeak^7^ in their 2017 article given the similar goal in estimating the economic impact of NAS. As such, we conducted matching and multiple regression to estimate long-term costs. We performed *t* tests on continuous variables and χ^2^ tests on categorical variables to assess the variation related to an NAS diagnosis.

Propensity score methods were used to match individuals with and without an NAS diagnosis based on sex, rurality, birth year, eligibility months, race and ethnicity, birth weight, gestational age, and the mother’s age at delivery. These variables were selected based on the study by Corr and Hollenbeak^7^ to account for cost variations based on demographic disparities and medical conditions likely to derive from other sources, particularly premature birth. Eligibility months helped control for variations in health care costs based on the enrollment period. As in the study by Corr and Hollenbeak,^7^ nearest-neighbor matching was used at a 1:1 ratio without replacement or caliper. Due to the confinement of the study population to Alabama and the similar socioeconomic status of participants given Medicaid enrollment, these elements were not included in matching.

Next, we performed multiple regression on the unmatched and matched samples to separate the respective costs of NAS over the term of eligibility. We used a generalized linear model assuming a γ distribution and a log link function. This model has become a standard tool in estimating health care costs due to the inherent skewness of these costs.^17,18^ Marginal effect estimates are reported to show the cost of each variable. To assess the cost distribution over the 10-year period, we aggregated paid amounts over the years following birth. We then performed *t* tests between the groups with and without NAS for years 1 through 10. A Bonferroni adjustment was used to avoid inflating type I error.

Analyses were conducted in June 2022 using Stata, release 16 (StataCorp LLC).^19^ Matching was performed using psmatch2 and regression using glm in Stata, release 16.^20^

## Results

Among 346 259 neonates with up to 10 years of Medicaid eligibility born in the state of Alabama from January 1, 2010, to December 31, 2020 (mean [SD] gestational age, 38.4 [2.2] weeks), our study identified 4027 born with NAS (1.2%) and 342 232 born without NAS (98.8%). The unmatched data showed significant differences between all variables used in matching. Mean (SD) Medicaid enrollment duration for individuals born without NAS was 60.0 (35.8) months and for those with NAS was 51.8 (32.1) months. Compared with the population without NAS, White individuals were significantly overrepresented in the population with NAS (69.4% vs 36.5%), and Black and Hispanic individuals were underrepresented (11.8% vs 39.0% and 1.7% vs 7.1%, respectively). Over the study period, the racial and ethnic breakdown of Alabama Medicaid births overall was 38.7%, Black; 7.0%, Hispanic; 36.8%, White; and 17.5%, other. Statewide birth estimates showed a breakdown of 31%, Black; 8%, Hispanic; 60%, White; and 2%, other.^21^ Males were also disproportionately represented in the population with NAS (45.2%, female; 52.7%, male; and 2.2%, unknown) compared with the population without NAS (48.2%, female; 50.5%, male; and 1.3%, unknown). These disparities are detailed in Table 1.

**Table 1.** Characteristics of Neonates With and Without NAS

Characteristic	Neonates^a^		Mean difference (95% CI)^b^
	Without NAS (n = 342 232)	With NAS (n = 4027)	
Race and ethnicity			
Black	133 386 (39.0)	474 (11.8)	−27.2 (−28.7 to −25.7)
Hispanic	24 319 (7.1)	70 (1.7)	−5.4 (−6.2 to −4.6)
White	124 765 (36.5)	2796 (69.4)	32.9 (31.8 to 34.1)
Other^c^	59 762 (17.5)	687 (17.1)	−0.4 (−1.6 to 0.8)
Sex			
Female	164 969 (48.2)	1819 (45.2)	−3.0 (−4.6 to −1.5)
Male	172 721 (50.5)	2121 (52.7)	2.2 (0.6 to 3.8)
Unknown	4542 (1.3)	87 (2.2)	0.9 (0.4 to 1.4)
Gestational age, mean (SD), wk	38.4 (2.1)	37.0 (3.2)	−0.4 (−0.5 to −0.4)
Birth weight, mean (SD), g^d^	3143.0 (579.2)	2931.0 (613.2)	−212.0 (−231.1 to −192.8)
Mother’s age at delivery, mean (SD), y	25.1 (5.4)	28.5 (5.1)	3.4 (2.6 to 4.2)
Year			
2010	32 272 (9.4)	207 (5.1)	−4.3 (−5.0 to −3.6)
2011	28 721 (8.4)	156 (3.9)	−4.5 (−5.1 to −3.9)
2012	28 896 (8.4)	211 (5.2)	−3.2 (−3.9 to −2.5)
2013	29 695 (8.7)	269 (6.7)	−2.0 (−2.8 to −1.2)
2014	28 923 (8.5)	272 (6.8)	−1.7 (−2.5 to −0.9)
2015	31 220 (9.1)	433 (10.8)	1.7 (0.7 to 2.7)
2016	29 155 (8.5)	450 (11.2)	2.7 (1.7 to 3.7)
2017	28 046 (8.2)	493 (12.2)	4.0 (3.0 to 5.0)
2018	29 152 (8.5)	514 (12.8)	4.3 (3.3 to 5.3)
2019	29 619 (8.7)	373 (9.3)	0.6 (−0.3 to 1.5)
2020	28 442 (8.3)	404 (10)	1.7 (0.8 to 2.6)
2021	18 091 (5.3)	245 (6.1)	0.8 (0.1 to 1.5)
Medicaid eligibility duration, mean (SD), mo	60.0 (35.8)	51.8 (32.1)	−8.2 (−9.3 to 7.1)
High-risk pregnancy	102 854 (30.0)	2095 (52.0)	22.0 (20.5 to 23.4)
Mother previously prescribed opioids	139 635 (40.8)	2642 (65.6)	24.8 (23.3 to 26.3)
NICU length of stay, mean (SD), d	2.5 (13.3)	16.2 (22.3)	13.7 (13.3 to 14.2)

Abbreviations: NAS, neonatal abstinence syndrome; NICU, neonatal intensive care unit.

^a^ Data are presented as the number (percentage) of neonates unless otherwise indicated.

^b^ Differences are expressed as percentages for categorical variables.

^c^ Includes all non-Hispanic races not listed.

^d^ For 291 409 neonates without and 3552 with NAS.

Neonates with an NAS diagnosis had a lower weight at birth (mean difference, −212.0 g; 95% CI, −231.1 to −192.8 g), and mothers of neonates with NAS were a mean of 3.4 years (95% CI, 2.6-4.2 years) older at delivery. While pregnancy risk status was not included in the matching criteria because of direct correlations with NAS, pregnancies for neonates with NAS were more likely to be high risk. Furthermore, the mean (SD) number of days spent in a neonatal intensive care unit (NICU) was higher for neonates with NAS compared with those without NAS (16.2 [22.3] vs 2.5 [13.3] days). The characteristics of the matched population were largely similar, with slight variations in race and ethnicity and birth year distributions. Table 2 also shows a large discrepancy in the mean (SD) number of NICU days at 15.9 (22.2) for neonates with an NAS diagnosis and 4.9 (18.0) for those without.

**Table 2.** Characteristics of Neonates With and Without NAS After 1:1 Matching

Characteristic	Neonates^a^		Mean difference (95% CI)^b^
	Without NAS (n = 3330)	With NAS (n = 3330)	
Race and ethnicity			
Black	397 (11.9)	410 (12.3)	0.4 (−2.0 to 1.2)
Hispanic	54 (1.6)	59 (1.8)	0.2 (−0.8 to −0.5)
White	2290 (68.7)	2268 (68.1)	−0.6 (−1.6 to 2.9)
Other^c^	589 (17.7)	593 (17.8)	0.1 (−2.0 to 1.7)
Sex			
Female	1532 (46)	1493 (44.8)	−1.2 (−1.2 to 3.6)
Male	1753 (52.6)	1766 (53.0)	0.4 (−2.8 to 2.0)
Unknown	45 (38.8)	71 (61.2)	22.4 (20.1 to 24.7)
Gestational age, mean (SD), wk	37.7 (3.0)	37.7 (2.3)	0.0 (−0.2 to 0.1)
Birth weight, mean (SD), g	2957.6 (643.0)	2944.3 (613.0)	−13.3 (−16.9 to 43.4)
Mother’s age at delivery, mean (SD), y	28.4 (6.1)	28.3 (5.1)	−0.1 (−0.0 to 0.3)
Year			
2010	204 (6.1)	198 (5.9)	−0.2 (−0.2 to −0.2)
2011	167 (5.0)	128 (3.8)	−1.2 (−1.2 to −1.19)
2012	210 (6.3)	167 (5.0)	−1.3 (−1.3 to −1.3)
2013	239 (7.2)	209 (6.3)	−0.9 (−0.9 to −0.9)
2014	1 (<0.1)	0	0.0 (−0.0 to 0.0)
2015	343 (10.3)	400 (12.0)	1.7 (1.7 to 1.7)
2016	361 (10.8)	420 (12.6)	1.8 (1.8 to 1.8)
2017	417 (12.5)	462 (13.9)	1.4 (1.4 to 1.4)
2018	385 (11.6)	480 (14.4)	2.8 (2.8 to 2.8)
2019	332 (10.0)	308 (9.2)	−0.8 (−0.8 to −0.8)
2020	402 (12.1)	346 (10.4)	−1.7 (−1.7 to −1.7)
2021	269 (8.1)	212 (6.4)	−1.7 (−1.7 to −1.7)
Medicaid eligibility duration, mean (SD), mo	51.9 (32.6)	51.1 (31.4)	−0.8 (−0.8 to 2.3)
High-risk pregnancy	1668 (50.1)	1690 (50.8)	0.7 (−3.1 to 1.7)
Mother previously prescribed opioids	2198 (66.0)	2188 (65.7)	−0.3 (−2.0 to 2.6)
NICU length of stay, mean (SD), d	4.9 (18.0)	15.9 (22.2)	11.0 (−12.0 to −10.0)

Abbreviations: NAS, neonatal abstinence syndrome; NICU, neonatal intensive care unit.

^a^ Data are presented as the number (percentage) of neonates unless otherwise indicated.

^b^ Differences are expressed as percentages for categorical variables.

^c^ Includes all non-Hispanic races not listed.

Table 3 shows estimates for an individual’s long-term health care costs based on variables from the model that were associated with significant overall cost differences. These variables included NAS diagnosis, gestational age at birth, birth weight, months of Medicaid eligibility, and whether the birth was via cesarean delivery. The reference population was individuals born without NAS who had a mean value for all considered variables. These individuals were expected to have a health care cost of $20 474 over 51.5 (32.0) months, the mean (SD) time of enrollment. The anticipated cost for an individual with NAS born vaginally at the mean (SD) gestational age (37.7 [2.6] weeks) and birth weight (2951.0 [628.1] g) was calculated by summing the constant ($20 474) with the amount for NAS ($17 921) to get an estimated cost of $38 395. This also assumed the average enrollment period. Each additional month of Medicaid enrollment was estimated to cost $126. The same individual would have an additional cost of $4504 if they were born 3 weeks earlier than the mean gestational age at birth, calculated by multiplying the cost associated with gestational age at birth (−$1501) by 3 and subtracting it from the total cost. Individuals with an NAS diagnosis had an estimated health care cost 2.3 times as much as those without. An NAS diagnosis accounted for an additional $17 921 (95% CI, $14 830-$21 012) in estimated paid amounts over 10 years.

**Table 3.** Marginal Effect Estimates From a Generalized Linear Model of 10-Year Cost

Variable	Cost (95% CI)^a^
Constant	20 474
NAS	
No	[Reference]
Yes	17 921 (14 830 to 21 012)
Gestational age at birth^b^	−1501 (−1748 to −1255)
Birth weight^b^	−7 (−8 to −5)
Months of Medicaid eligibility^b^	126 (84 to 168)
Cesarean delivery	
No	[Reference]
Yes	7311 (4334 to 10 288)

Abbreviation: NAS, neonatal abstinence syndrome.

^a^ In 2021 US dollars.

^b^ For the reference population, it was assumed that the neonate would not have NAS. If the infant did have NAS, the cost would increase by an estimated $17 921.

We were also interested in how this cost was distributed over time. Given that $18 000 of total costs can be associated with NAS and $12 000 can be attributed to the initial hospital stay,^7^ we inferred that NAS would account for long-term spending. Significance tests showed that individuals with NAS had higher costs in years 1 (mean difference, $21 159; 95% CI, $20 251-$24 067), 2 (mean difference, $2253; 95% CI, $1256-$3251), 5 (mean difference, $873; 95% CI, $164-$1582), 7 (mean difference, $1559; 95% CI, $829-$2289), and 8 (mean difference, $1397; 95% CI, $465-$2328). These higher costs likely correlate with children starting school, where additional health and development problems can be discovered.^22^ Table 4 depicts cost differences across the first 10 years of life.

**Table 4.** Distribution of NAS-Related Costs by Year of Life^a^

Year of life	Yearly cost without NAS, mean $ (95% CI)	Individuals, No.	Yearly cost with NAS, mean $ (95% CI)	Individuals, No.	Mean difference, $ (95% CI)
1	11 727 (11 513-11 943)	417 365	33 886 (31 659-36 112)	5421	22 159 (20 251 to 24 067)
2	3260 (3149-3370)	332 694	5513 (3608-7418)	4291	2253 (1256 to 3251)
3	2360 (2286-2434)	277 751	2918 (2569-3267)	3451	558 (−106 to 1221)
4	2212 (2146-2279)	204 889	2699 (2275-3123)	2404	487 (−128 to 1102)
5	2305 (2228-2381)	198 193	3177 (2432-3922)	2344	873 (164 to 1582)
6	2358 (2267-2449)	169 249	3173 (2592-3754)	1888	815 (−49 to 1679)
7	2488 (2414-2561)	142 444	4047 (3144-4949)	1474	1559 (829 to 2289)
8	2572 (2482-2662)	116 843	3969 (2582-5355)	1115	1397 (465 to 2328)
9	2702 (2591-2813)	94 1222	3855 (2336-5373)	829	1152 (−38 to 2343)
10	2672 (2535-2809)	72 440	3151 (2058-4245)	581	479 (−1056 to 2014)

Abbreviation: NAS, neonatal abstinence syndrome.

^a^ Costs are in 2021 US dollars.

## Discussion

To our knowledge, this is the first study to use data more recent than 2014 in examining long-term costs of NAS and the first to link records of mothers and neonates. Given that Alabama has the highest opioid prescribing rates in the country^10^ and a rising incidence of NAS,^4^ understanding of the long-term costs of NAS in a worst-case scenario allows for better planning and management of resources. Rising NAS incidence also underscores the importance of current data. Between 2010 and 2020, the percentage of neonates born to mothers covered by Alabama Medicaid increased from 0.6% to 2.0%.^9^ These individuals also represent a disproportionate percentage of costs, with neonates with NAS accounting for 3.3% of first-year infant payments in 2020.^9^ Our ability to link data from mothers and neonates adds a layer of credibility through more actionable insights. Furthermore, there is a high density of NAS cases in the southeastern US, where medical costs and the cost of living are similar to those in Alabama.^2^

Examining the Medicaid population is crucial given that state Medicaid agencies^3,7^ cover approximately 80% of individuals born with NAS. This demographic profile of individuals in this study is comparable to that of the population in the existing literature. As in studies by Winkelman et al^6^ and Corr and Hollenbeak,^7^ the population with NAS in the current study was mainly White and males were overrepresented. The incidence of NAS in the US population is also rising.^5^ This is not surprising given the rise in maternal opioid use.^2^

In Alabama, women receiving Medicaid benefits because of pregnancy are automatically enrolled in the Alabama Coordinated Health Network (ACHN) program.^23^ In response to the increase in substance use disorder and infant mortality, ACHN entities screen pregnant women for opioid use as part of the case management system. The fiscal implications of the current study suggest that neonates with an NAS diagnosis may also need to be enrolled in case management to manage long-term costs and address developmental delays before they reach the age to begin school.

Not all health care costs should be viewed as negative; use of services can lead to early-stage diagnoses or prevention of health conditions. As such, Medicaid members with no health care costs are a major concern. Most state Medicaid agencies report health statistics such as well-child visits to the Centers for Medicare & Medicaid Services. This indicates the importance of these visits to prevent additional health care utilization and costs at a later date.^24^

The current study’s findings suggest that NAS has health consequences extending to adolescence and long-term fiscal outcomes for public insurance agencies beyond the first year of life. This study is an important step in elucidating the consequences of NAS for the US health care system. Future studies should examine the cost and long term impact of NAS in other US Medicaid populations. Under this study’s model, assuming the reference characteristics of the study population, NAS will account for $3.8 million over 10 years in spending incurred by the 212 individuals born with NAS to Medicaid-eligible mothers in Alabama in 2021. These costs could be avoided with intervention in the lives of expectant, Medicaid-eligible mothers. Given recent opioid-related lawsuits and settlements, $50 billion will be made available to various states and localities.^25^ As administrative structures are implemented to distribute this money, interventions should be considered to combat the occurrence of NAS and reduce associated costs.^25^

### Limitations

This study has limitations. Identifying individuals born with NAS relies on a timely, adequately documented claim. Individuals could have NAS without an identified *ICD-9* or *ICD-10* code on record, or they could be diagnosed more than 30 days after birth. Propensity score matching mitigated the effects of included confounders. However, as seen in the difference in NICU days between the prematched and matched samples, the selected neonates without NAS in the matched sample needed more initial care than did those in the prematched sample. Additionally, the matching criteria may not account for all variation. As mentioned, 2014 birth weight data were missing, and these neonates were excluded from the matched sample. The costs incurred by individuals born with NAS may also vary across states and insurance programs due to unique policies and programs.

Furthermore, the environment for individuals with vs without a previous NAS diagnosis can vary widely, as socioeconomic status and unemployment are associated with opioid use.^26^ Health outcomes are affected by a child’s environment, and women with opioid use disorders are more likely to experience mental health conditions, have a history of abuse, and lack support systems.^27,28,29^ These factors could also account for increased medical costs.

## Conclusions

In this cohort study of neonates born into the Alabama Medicaid population, those with an NAS diagnosis had a different demographic profile than those without NAS and incurred higher costs to state Medicaid agencies. These findings suggest that further efforts are warranted to reduce the occurrence of NAS.

## Notes

Supplement.
Data Sharing Statement
