# Supplementary material for: Cost of Care for Newborns With Neonatal Abstinence Syndrome in a State Medicaid Program
Source: JAMA Netw Open. 2024 Feb 26;7(2):e240295. doi: 10.1001/jamanetworkopen.2024.0295 (PMC10897737; doi:10.1001/jamanetworkopen.2024.0295)
Supplement: Supplement. — Data Sharing Statement [file jamanetwopen-e240295-s001.pdf]

Data Sharing Statement

Jenkins. Cost of Care for Newborns With Neonatal Abstinence Syndrome in a State Medicaid Program. *JAMA Netw Open*. Published February 26, 2024.
doi:10.1001/jamanetworkopen.2024.0295

Data
Data available: No

Additional Information
Explanation for why data not available: Data will not be made available due to PHI concerns.
